# Polyurethane derived from castor oil monoacylglyceride (*Ricinus communis*) for bone defects reconstruction: characterization and in vivo testing

**DOI:** 10.1007/s10856-021-06511-z

**Published:** 2021-04-01

**Authors:** João Pedro Pereira de Morais, Isnayra Kerolaynne Carneiro Pacheco, Antonio Luiz Martins Maia Filho, Daniel Cabral Leão Ferreira, Felipe José Costa Viana, Fernando da Silva Reis, José Milton Elias de Matos, Marcia dos Santos Rizzo, Ana Cristina Vasconcelos Fialho

**Affiliations:** 1 Graduated Program in Health Sciences, Health Sciences Center, Federal University of Piauí, Ininga Campus, Teresina, Brazil; 2 Department of Physiology, Health Sciences Center, State University of Piauí, Teresina, Brazil; 3 Department of Chemistry, Nature Sciences Center, Federal University of Piauí, Ininga Campus, Teresina, Brazil; 4 Department of Morphology, Health Sciences Center, Federal University of Piauí, Ininga Campus, Teresina, Brazil; 5 Department of Pathology and Dental Clinic, Health Sciences Center, Federal University of Piauí, Ininga Campus, Teresina, Brazil

## Abstract

Biomaterials used in tissue regeneration processes represent a promising option for the versatility of its physical and chemical characteristics, allowing for assisting or speeding up the repair process stages. This research has characterized a polyurethane produced from castor oil monoacylglyceride (*Ricinus communis* L) and tested its effect on reconstructing bone defects in rat calvaria, comparing it with commercial castor oil polyurethane. The characterizations of the synthesized polyurethane have been performed by spectroscopy in the infrared region with Fourier transform (FTIR); thermogravimetric analysis (TG/DTG); X-ray diffraction (XRD) and Scanning Electron Microscopy (SEM). For the in vivo test, 24 animals have been used, divided into 3 groups: untreated group (UG); control group treated with Poliquil® castor polyurethane (PCP) and another group treated with castor polyurethane from the Federal University of Piauí - UFPI (CPU). Sixteen weeks after surgery, samples of the defects were collected for histological and histomorphometric analysis. FTIR analysis has shown the formation of monoacylglyceride and polyurethane. TG and DTG have indicated thermal stability of around 125 °C. XRD has determined the semi-crystallinity of the material. The polyurethane SEM has shown a smooth morphology with areas of recesses. Histological and histomorphometric analyzes have indicated that neither CPU nor PCP induced a significant inflammatory process, and CPU has shown, statistically, better performance in bone formation. The data obtained shows that CPU can be used in the future for bone reconstruction in the medical field.

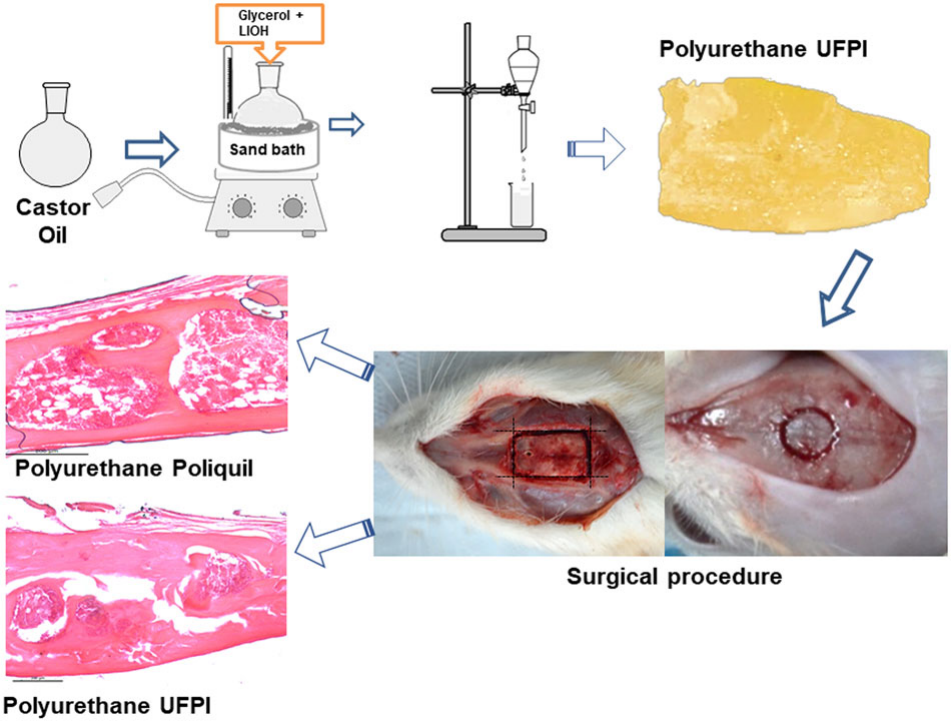

## Introduction

Bone defects commonly caused by trauma, congenital deformities, or inflammatory diseases still represent a challenge for orthopedics, oral and maxillofacial surgery, with bone tissue engineering having the aim of helping in their reconstruction [1, 2]. For this purpose, it is necessary to use biomaterials that accelerate or assist the standard and complete repair of the defect [3].

The research for biomaterials produced from raw matter extracted from nature to promote healing and new bone formation has grown in recent years. Vegetable oils, formed mainly by triglycerides, provide excellent raw material for the synthesis of polymeric materials because of their low toxicity, low production and processing cost, and because they are generally biodegradable [4]. Therefore, some polyurethanes have emerged as materials for biological implants or grafts because of their mechanical properties, chemical stability, and biocompatible nature [5, 6]. In this context, with polyurethanes, tissue engineering has a relevant role in searching for absorbable materials that can improve health care systems’ quality, with their formulations that allow creating organs, tissues, implants, and grafts. Castor oil-based polyurethanes have been widely explored for biomedical devices and tissue engineering applications, thanks to their qualities such as good biocompatibility, biodegradability, naturally occurring hydroxyl groups, and easy chemical modification. However, many properties and characteristics are required for medical devices and tissue engineering, such as good mechanical performance, a 3D structure, and a degradation rate compatible with the application. Various methods can circumvent these barriers, from the combination of chemical modification techniques, adapting the device’s properties, suitable formats, or variations in the polyol-isocyanate composition [6–9]. Polymers are macromolecules with high molar mass, in the order of 103–106 g/mol, formed by the repetition of chemical units called “mere”. The classification of polymers is based on functional groups, such as polyamide, polyester, polyether, polyurethane, and polyacrylate [6]. Polyurethane (PU) is a polymer that contains urethane groups in its main chain. The structure of urethane results from a chemical reaction between an isocyanate group and a hydroxyl group. The synthesis of polyurethane results from the reaction between a compound with two or more isocyanates and polyfunctional alcohol (hydroxylated low molecular weight polymer or polyol) [7–9].

Granule-shaped polyurethanes have been investigated to influence granule size on the resorption process and the ability to promote bone healing. The granules of the Poliquil® Ricinus Bone compound correspond to sizes of 450 µm, while the polyurethane produced at UFPI was reduced to granules of medium size of 370 µm, important data to be considered for the action on the bone regeneration process. The 450 µm castor oil polyurethane particles allow the metabolism and growth of osteogenic cells that will take part in the formation of new bone tissue [10]. Tiny particles may not leave enough space to allow the migration and internal growth of cells, blood vessels, and bones. The particle size of about 380 µm produces minimal interparticular space or pores large enough for adequate vascularization and bone formation [11]. Polyurethanes derived from castor oil have a molecular formula compatible with living tissues, excellent structural properties, besides not releasing toxic vapors and radicals when implanted and having low cost.

For this reason, it has presented excellent reports on its in vivo behavior after implantation in bone tissue [9–13]. The properties of PUs prepared with castor oil and monoacylglyceride from castor oil may have different properties, such as greater or lesser rigidity of the chain, resulting from the rigid and flexible segments, related to the size of the chain, and this can affect crystallinity, mechanical strength, among other PU properties. However, in this study, we did not intend to explore the differences between castor oil and its monoacylglyceride. This research aimed to characterize a polyurethane produced from the castor oil monoacylglyceride (Ricinus communis L.) and test its effect on the bone regeneration process in rats with calvaria defects.

## Materials and methods

### Reagents and materials

The pure Castor oil was obtained in a specialized store (mundodosoleos.com.br). The reagents used in the production of monoacylglycerides (MAG) and its polymer (CPU) were glycerol (C_3_H_8_O_3_, Impex), lithium hydroxide (LiOH, Vetec), and Hexamethylene Diisocyanate -HDI (C_8_H_12_N_2_O_2_, Sigma-Aldrich) for polymerization.

### Polyurethane synthesis from MAG from castor oil

We have obtained the MAG and polyurethane as described by Cunha and coworkers [14], Moura Neto and coworkers [15], and Nascimento and coworkers [16].

The triglycerides glycerolysis reaction, present in the chemical structure of castor oil, started MAG synthesis, done in a 100 mL round bottom flask at ~140 °C in a stirred sand bath for 5 h; after stabilizing the system temperature, the reagents (oil and glycerol) were added to the flask and subjected to stirring; then the catalyst, LiOH, was added. The number of reagents followed the 1: 4 M ratio (oil: glycerol), and the amount of LiOH corresponded to 0.05% of the oil mass. At the end of the reaction, a separation funnel was used to collect the formed MAG. After stabilizing the temperature at about 130 °C in a similar system, HDI was gradually added to the previously obtained MAG. The reaction lasted for 5 h and occurred in the absence of solvent, following a stoichiometric ratio of 1: 4 (MAG: HDI). The ratios used here have been chosen based on our previous studies [14–16], in which a broader review was possible.

Poliquil® (Araraquara, Brazil) kindly provided the granulated castor polyurethane for the experiment.

### Characterizations

The FTIR spectra of MAG and polyurethane samples were obtained in a Thermo Fisher SCIENTIFIC NICOLET iS5 spectrophotometer with a purge pump and wavelength between 400 and 4000 cm^−1^, 128 accumulated scans, 4 cm^−1^ resolution, in ATR, in Transmittance module. The method has been used to verify the curing reaction of polyurethane and free isocyanate functional groups (NCO) after the response [14–17]. The TG/DTG curves were obtained simultaneously in the SD Instruments Q 600, from TA Instruments, using a heating rate of 10 °C/min, between 25 °C and 600 °C, in N2 atmosphere, with a sample mass of ~10 mg [14]. Polyurethane samples were analyzed for their microstructure using X-ray diffraction (XRD), Labx - XDR 600 equipment from Shimadzu with Cu-Kα radiation (λ = 1.5406 Å), 2θ in the range between 50° and 75°, the scan rate of 2°/min and total exposure time of 40 min. The objective was to identify crystalline peaks, crystalline, and amorphous phases, and also calculate the parameters of crystalline structure of the biomaterial [14, 18]. To perform Electron Microscopy, FEI Quanta 250 FEG-equipment has been used. Because it was a polymeric material, surface preparation has been necessary. All samples were fractured in liquid nitrogen and coated with a gold layer to obtain better conductivity. A piece was deposited in the aluminum substrates. For this purpose, the samples have been coated with gold to avoid cartoon accumulation that repels the incident electron beam. The micrographs can be used to observe the morphology of polyurethane [14].

### In vivo testing

Twenty-four adult male rats (*Rattus norvegicus*, Wistar) have been used, randomly divided into 3 groups (*n* = 8), according to the material used or not for calvaria reconstruction: untreated group (UG), control group treated with Poliquil® castor polyurethane (PCP) and castor polyurethane UFPI (CPU). The latter polyurethane has been produced at the Materials Physics Laboratory (FISMAT) at the Federal University of Piauí (UFPI). This research has been approved by the local Animal Use Ethics Committee (CEUA), protocol nº 00850/2016. The procedures performed in this study have followed the ethical principles established by the National Council for the Control of Animal Experimentation (CONCEA) and the National Institutes of Health guide (NIH) [19, 20].

The animals were anesthetized with xylazine (10 mg/kg) (0.2% xylazine hydrochloride - Virbaxyl®) [17, 19]. After trichotomy of the surgical area, a craniocaudal incision was made in the midline, exposing the calvaria where a 6 mm diameter defect was made in the central region, with the aid of a trephine drill (Harte Precision Grip®) and low -rotation, under constant irrigation [20]. The defects were filled with polyurethanes, according to the group to which each animal belonged. The CPU was ground to a granule shape and subjected to the autoclave sterilization process. The periosteum and the skin were sutured with 5.0 needle nylon suture thread (Shalon surgical wire Ltd®). The animals received analgesia with tramadol (2 mg/kg), by deep intramuscular route, every six hours, 24 h postoperatively, and were euthanized after 16 weeks. A rectangular portion of the calvaria containing the defect was removed for histological processing.

#### Qualitative histological evaluation

The pieces were fixed in 10% neutral buffered formalin solution, decalcified in 30% formic acid solution (CH_2_O_2_, Sigma-Aldrich), and 20% sodium citrate (Na_3_C_6_H_5_O_7_, Sigma-Aldrich) (1:1). Before conventional histological processing for paraffin embedding, which constitutes the processes of dehydration, clarification, paraffin embedding, packaging, and microtomy. Serial sections of 5.0 µm thick were obtained, adhered to glass slides, subjected to the process of diaphanization (dewaxing) and rehydration in xylol and alcohol, and stained histochemically with hematoxylin-eosin (H.E.) and Masson’s Trichrome (MT) for histological analysis and histomorphometric evaluation. Qualitative histological analysis has been performed under light microscopy (Olympus BX51) by a pathologist who did not know which group the slides belonged to. The decalcified sections were observed with an objective lens at 10× magnification.

#### Histomorphometry

Histomorphometric analysis (blind) was performed to quantify the healing response from the samples. The defect margin was identified, and any newly formed bone was quantified by measuring the area of bone nucleation sites in each section and calculating the average total area of these sites by groups. Three non-consecutive images of each specimen section (*n* = 8) were photo-documented. The images were used for histomorphometric measurements using the ImageJ image analysis software (free version - imagej.nih.gov/ij/download/). The data were arranged in a table using Microsoft® Excel 2016 and analyzed statistically with SPSS 20.0 software (SPSS Inc., Chicago, IL, USA) for Windows.

### Statistical analysis

All quantitative data has been presented as mean and standard deviation. For histomorphometry data, normality analysis has been initially performed with the Shapiro–Wilk test, the analysis of variance test (One-way ANOVA) has been applied using the SPSS 20.0 software (SPSS Inc., Chicago, IL, USA) for Windows. Tukey HSD post-test was used to compare different scaffolds with the control (empty defect). Statistical differences were significant when *p* < 0,05.

## Results

### Castor bean PU synthesis

The production of polyurethane started with the synthesis of MAG from castor oil. HDI was added to MAG, resulting in the polymerization by isocyanate or prepolymer groups, which reacted to a polyol forming the polyurethane with a solid structure and recesses in the surface.

### Characterizations

**Fig. 1:**
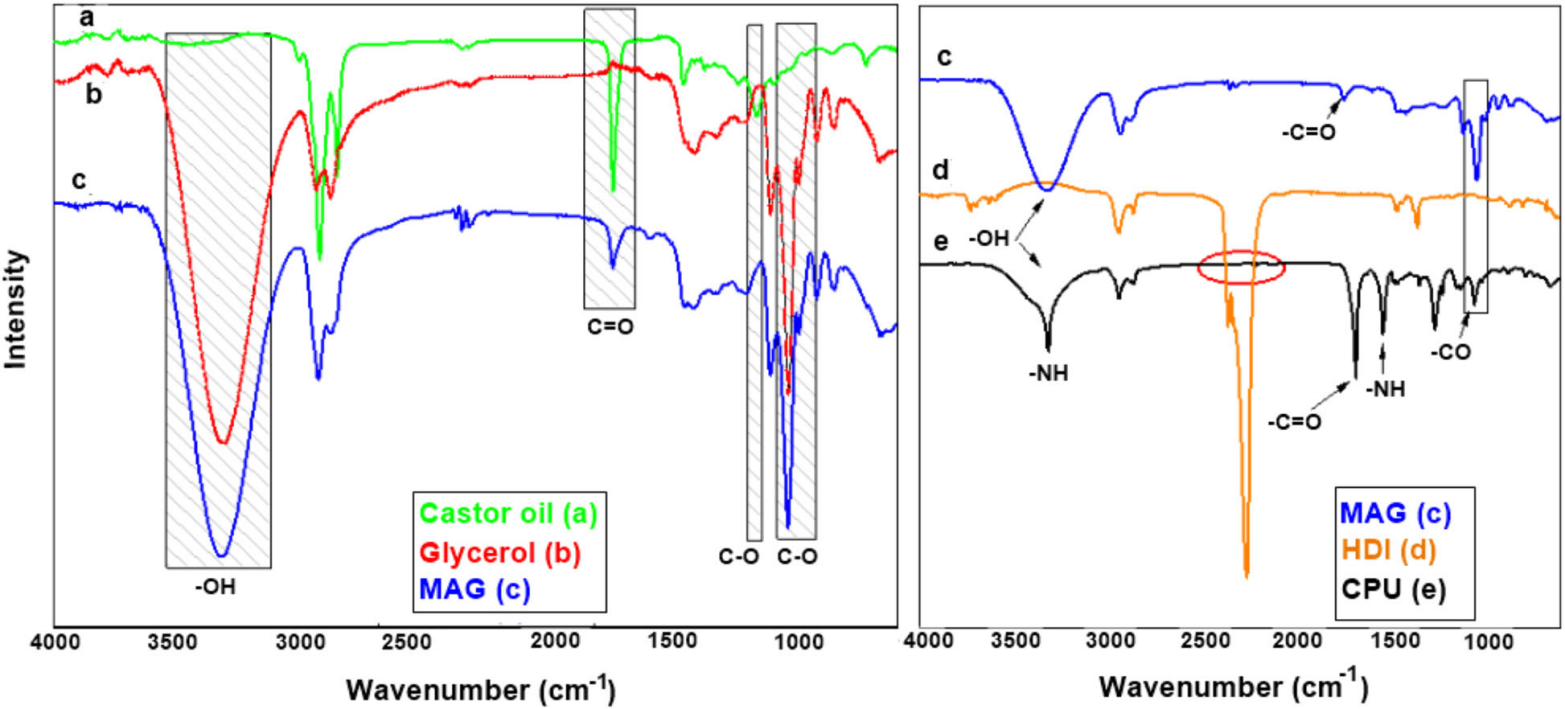
FTIR spectra: **a** castor oil, **b** glycerol, **c** MAG; **d** HDI and **e** CPU

The FTIR of MAG, obtained from castor oil (CO) and polyurethane, is observed in Fig. 1a, b a band at 3300 cm^−1^ is noted, which corresponds to the stretching vibration of OH groups present in glycerol and MAG. Bands at 2850 and 2930 cm^−1^ are assigned to the asymmetric and symmetric stretch of aliphatic –C–H. A band at 3014 cm^−1^ is related to the vibrational stretching of =C–H of alkenes. The band at 1168 cm^−1^ corresponds to ester connections (–C–O–C–), while the band at 723 cm^−1^ denotes asymmetric angular deformation of all groups –CH_2_–O–. Peaks of carbonyl group C=O of castor oil are shown in MAG (Fig. 1c) between 1739 and 1736 cm^−1^, which does not appear in the glycerol spectrum. The absorption bands at 1241 cm^−1^ and 1047 cm^−1^ denote C–O and C–C coupling, present in glycerol and monoglyceride [15]. Highlighted in Fig. 1e, it is possible to note regions that prove the polymer’s formation, as a characteristic band of the N–H urethane bond is found in 3300 cm^−1^. It is also observed in the CPU spectrum that the stretch band C=O shifted to 1678 cm^−1^ and that the stretch band attributed to the connection –C (O) O– (urethane) is found in 1234 cm^−1^.

The characteristic urethane band (NCO) (Fig. 1d), at 2262 cm^−1^, is not found in the CPU spectrum (Fig. 1e), confirming the total reaction between HDI and MAG [17, 18, 20].

**Fig. 2:**
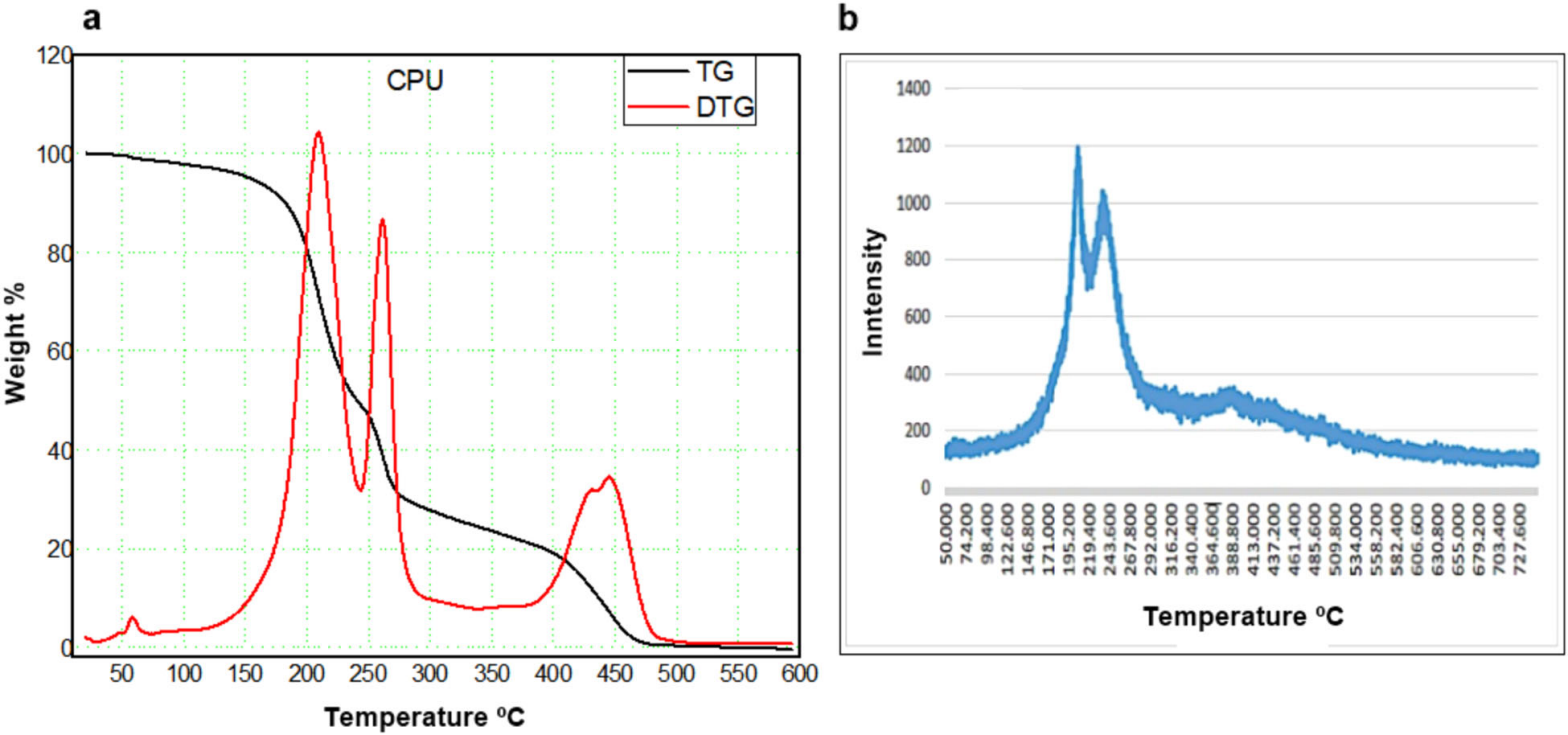
**a** TG and DTG curves for CPU polyurethane; **b** Diffractogram of polyurethane with crystallinity peak around 195.2 °C

Thermal analysis (TG and DTG) has shown thermal stability up to 125 °C and mass loss of the initial sample around 60 °C, which can be attributed to the evaporation of reagent residues or oligomers, Fig. 2a. The absence of characteristic phase transformation peaks suggests the predominance of the material amorphous phase. Two peaks observed in DTG coincide with the mass loss present in TG between 175 °C to 225 °C and 225 °C and 275 °C. A gradual mass loss above the latter is related to the decomposition of long polyurethane chains [15, 16].

X-ray diffraction (XRD) (Fig. 2b) presents a diffractogram with peaks around 195.2º, 243.6°, and 388.8°, indicative of semicrystalline polymer. The fact that the bonds cause an orderly arrangement of atoms or molecules with regular repetition in space can explain this semi-crystallinity. The molecular chains are densely packed in an ordered and parallel configuration, which justifies that the addition of HDI to castor oil leads to the formation of urethane groups through bonds between HDI and hydroxyls (–OH) [16–18, 20].

**Fig. 3:**
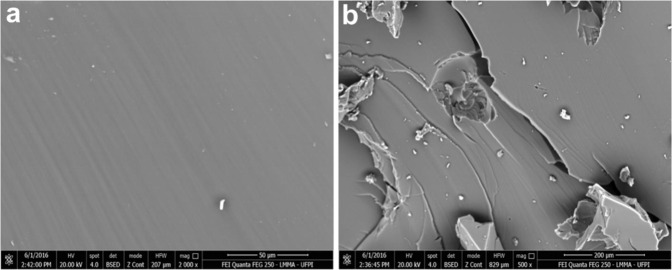
SEM images of castor/UFPI polyurethane sample; **a** flat, regular and continuous surface, with no pores (2000×); **b** homogeneous surface with protrusions and depressions, fracture and artifacts (500×)

SEM has provided data on the polyurethane microstructure, covering the material surface topography, characterized by being flat, homogeneous, with sparse grooves and lighter structures, suggestive of artifacts by residual material (Fig. 3).

### Qualitative histological evaluation

**Fig. 4:**
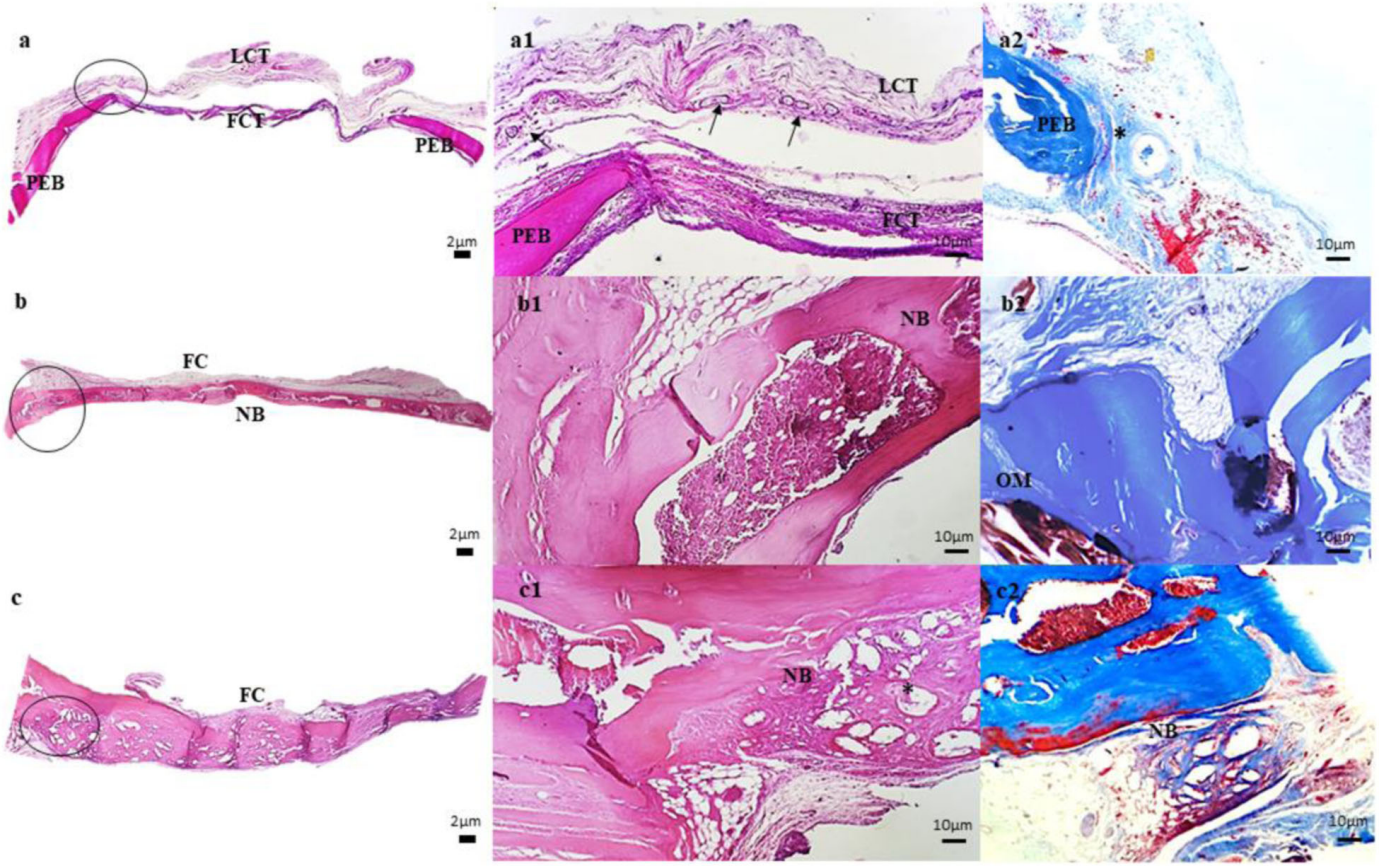
Photomicrographs of bone repair in a critical rat calvaria defect, after 16 weeks, in UG group (**a**, a1, and a2), PCP group (**b**, b1, and b2), and CPU group (**c**, c1, and c2). FC fibrous capsule, FCT fibrous connective tissue, LCT loose connective tissue, NB newly formed bone, OM osteoid matrix, PEB pre-existing bone; *polyurethane particle. H.E. Hematoxylin-Eosin, MT Masson’s trichrome. Scale bar: 2 and 10 µm

Qualitative histological examination on the H.E. staining slides showed, in the UG group (Fig. 4a), a predominance of loose and very vascularized connective tissue covering the entire area of the defect (Fig. 4a1–black arrows). There are moderate fibrous connective tissue, and individualized collagen fibers connecting the two pre-existing bone tissue ends in its most central portion. Newly formed bone tissue is not present in these extremities, but most of them are anchored by a connective tissue containing a layer of slightly denser collagen fibers and greater deposition of the extracellular matrix.

The PCP group (Fig. 4b) showed a newly formed bone tissue covered by a fibrous connective tissue capsule along the entire length of the defect, demonstrating a trabecular structure with lamellations, compatible with physiological consolidation of secondary bone marrow (Fig. 4b1). An osteoid matrix with fibers was observed. There were fragments of the polymer in some samples not yet absorbed but with no apparent granulomatous inflammatory reaction.

In the CPU group (Fig. 4c), the formation of thick osteoid lamellae was observed, associated with multiple areas of a fibrous structure still discernible. This newly formed spongy bone trabecula (primary bone lamella) fills the entire area of the defect and anchors itself to the pre-existing and more mineralized bone tissue, located more externally to the defect. Besides, there were no signs of bone anomalies or bone resorption around the defect site, and the CPU scaffold was almost completely degraded and replaced with new tissue.

### Histomorphometry

Quantitative histological evaluations were performed on the samples to assess bone formation levels in the critical defect site. Thus, it was observed that the UG group and the CPU group had, respectively, the lowest and highest mean of newly formed bone tissue, according to Table 1. The variance analysis has shown a difference between the groups (*p* < 0.01), and the Tukey HSD post-test has shown a significant difference between the UG, PCP, and CPU groups. At 120 days post-implantation, the newly formed bone area in the critical defect treated with the CPU scaffold was ~3.6 and 0.52 times larger than untreated empty defect and PCP controls, respectively.

**Table 1:** Comparing the means and standard deviation (SD) of each group’s newly formed bone area (*n* = 8)

	Control	Poliquil® castor polyurethane	Castor polyurethane UFPI	p*
Neoformed bone tissue area (mm^2^)	Mean (SD) 0.52 (±0.26)^a^	Mean (SD) 1.58 (±0.33)^b^	Mean (SD) 2.40 (±0.61)^c^	<0.01

Tukey post-test represented by different lowercase letters (a, b, c) on the same line, indicating a significant difference

*statistically significant for one-way ANOVA (*p* < 0.01)

## Discussion

A polyurethane was produced from castor monoacylglyceride (UFPI) and, to confirm its production and evaluate its performance, characterization tests and in vivo tests were performed.

It is relevant to compare the syntheses of polyurethanes used in this study, considering that they were produced with some variations of compounds and methods, which leads to changes in their properties. Polymers’ properties are mainly due to intermolecular and intramolecular interactions, the quantity, molar ratio, and functionality of the isocyanate and polyol used in their production [21]. The polyurethane synthesis produced at UFPI, confirmed by FTIR spectra, occurred through the glycerolysis reaction of the triglyceride present in the structure of castor oil to which the polyol (glycerol) was added. The catalyst (lithium hydroxide), with controlled time and temperature, monoacylglyceride (MAG) was obtained. Hexamethylene diisocyanate (HDI) was added to the monoacylglyceride to obtain polyurethane after the curing process occurred. HDI was chosen because it is less toxic and does not contain benzene, and contributes to improving its properties [15]. Obtained by a different method, the polyurethane produced by Poliquil® is marketed under the name of Ricinus Bone Compound (COR). Leonel et al. [17] describe that the synthesis took place from a diisocyanate (Diphenylmethane diisocyanate/MDI) reaction with a polyol derived from castor oil for the synthesis of the prepolymer. The polymerization occurred by the reaction between the prepolymer and polyol in a ratio of 1:0.65 of prepolymer and polyol, with the addition of calcium carbonate, considered a biocomponent reaction.

The use of different diisocyanates influences the rigid segment of polyurethanes, responsible for properties such as hardness, tensile strength, and toughness [21]. Sousa et al. [10] performed FTIR of granulated Poliquil® castor polyurethane, showing similar results to those found in this study, such as the OH stretch vibration bands at 3323 cm^−1^ and symmetrical and asymmetric stretch bands in the region 2925 and 2854 cm^−1^ corresponding to the CH_3_ group. Similar results of a polyurethane FTIR were found by Zhang et al. [18] when investigating the effects of the molar ratio of castor oil polyols on polyurethane structure and properties, noting that hydrogen bonds affect them [13–18, 20, 22]. The polyurethane of the company Poliquil® has excellent structural properties, which gives it compatibility with living tissues, does not release vapors and toxic radicals when implanted [17]. Similar data is given to polyurethane produced at UFPI, based on this research results, a pioneering study with this product, since it is biocompatible, osteoconductive, and osteointegrable. The results found in the FTIR characterization are corroborated by studies that confirm PU production.

In the TG curve of castor oil polyurethane (PU), thermal stability around 125 °C, with a reduction in the initial sample mass around 60 °C, observed in the DTG, probably resulting from the physical desorption of volatile components [15–23]. Moura-Neto et al. [15] state that because of their structural `characteristics, polyurethanes decompose in two or three stages; the first stage is related to the breakdown of the isocyanate and alcohol bond; the second is due to the decomposition of the polyol, and the third is because of the elimination of CO2. By TG and DTG, the degradation was evidenced in the following steps: between 60 °C and 175 °C with loss of mass around 10%; 175 °C and 275 °C loss of 60% of mass; 275 °C and 475 °C, remaining mass. Above 475 °C the organic residues underwent thermolysis [10, 15, 16, 23–26].

The morphometric analysis aims to test and subsidize a new therapeutic modality’s effectiveness in monitoring bone neoformation [22, 27–35].

Sousa et al. [10] evaluated the polyurethane produced by Poliquil® as a graft for treating bone defects in 16 rabbit rabbits, 8 animals were treated with polyurethane (treated), and 8 had the defects filled only by blood clots (control). In the same study, they carried out a second experiment, where they produced segmental defects in the zygomatic arch of 16 rabbits, 8 animals treated by guided bone regeneration, with latex membrane associated with polyurethane grafting, and 8 were not treated (control). The first experiment resulted in a greater bone volume in the treated group, 60 days after the operation. In the second experiment, bone bridging occurred in the defects treated at 60 and 120 days, different from the control group that presented incomplete healing. The authors concluded that Poliquil® polyurethane is biocompatible, osteoconductive, and osteointegrable. Leonel et al. [17] evaluated the action of the castor polymer during bone neoformation, using 45 rats, produced bone defects near the zygomatic arch of all animals, filling the defects with castor polymer blocks whose ends were fixed to the bone stumps with material resinous also derived from castor oil. After 15, 30, 60, 90, and 120 days, the animals were euthanized, and the pieces were processed for histological analysis. The results showed that the castor polymer helped the healing process, concluding that the castor polymer acted as an osteoconductor. These data corroborate the results found in this study.

Another important factor to be considered is the size of the polyurethane granules. Tiny particles can be used due to their fast absorption, greater surface area, and good osteogenesis. However, they may not leave enough spaces between them for passaging cells and blood circulation into the vessels and bone. A minimum space of over 100 µ is necessary to allow good vascularization and bone regeneration. Particles with a size of around 380 µ in diameter are indicated for producing a space between the particles considered desirable [11]. These data corroborate those of this study, where particles between 370 and 450 µm were used due to the use of UFPI polyurethane in orthopedic treatments. The histomorphometric analysis has shown better statistical results for the polymer of the CPU group. The difference concerning the UG and PCP group shows the possibility of using the polyurethane produced as a treatment for bone defects, as it has presented a significant area of bone formation. Therefore, the CPU scaffold showed many suitable properties, such as being biocompatible, degradable, non-toxic, and osteointegratable, besides a promising osteoconductive for bone regeneration.

The polyurethanes used have great relevance regarding the medical science of biomaterials, especially in the cost-benefit ratio of these products as they are low-cost materials.

## Conclusion

Histomorphometric characterizations and analyses show that the polyurethane obtained in our laboratory (CPU) has biocompatibility and behaves as an efficient osteoconductive and osteointegrating material, not inducing the formation of foreign body granulomas after 16 weeks succeeding surgery. Therefore, this polyurethane becomes a strong alternative for future bone defect regeneration in the medical field.
